# Supplementary material for: Extrahepatic Recurrence After Surgical Resection of Hepatocellular Carcinoma Without Intrahepatic Recurrence: A Multi-Institutional Observational Study
Source: Cancers (Basel). 2025 Apr 23;17(9):1417. doi: 10.3390/cancers17091417 (PMC12070905; doi:10.3390/cancers17091417)
Supplement: Supplementary file 1 [file cancers-17-01417-s001.zip › cancers-3566444-supplementary.pdf]

Supplementary Tables

Supplementary Table S1. Baseline characteristics of patients at initial HCC diagnosis (n = 1066)

Characteristics	Patients without recurrence (n = 531)	Patient with IHR (n=373)	Patients with IHR and EHR (n=124)	Patients without IHR at EHR (n = 38)	*P-value*
Age (years)	59.87 ± 10.22	59.13 ± 10.27	55.74 ± 9.77	58.94 ± 9.77	< 0.001
Male (n, %)	441 (83.1)	332 (89.0)	107 (85.6)	35 (92.1)	0.053
BMI (kg/m^2)	24.28 ± 3.14	23.80 ± 3.17	23.63 ± 2.93	24.16 ± 2.56	0.063
Etiology of cirrhosis, n (%)					0.665
HBV	321 (74.7)	242 (74.0)	87 (75.0)	26 (76.5)	
HCV	25 (5.8)	30 (9.2)	12 (10.3)	5 (14.7)	
Alcoholism	85 (19.8)	55 (16.8)	17 (14.7)	3 (8.8)	
Child-Pugh Score, n (%)					
A / B	503 (98.8) / 6 (1.2)			37 (97.4) / 1 (2.6)	
ALBI grade ≥ 2, n(%)	56 (10.6)			7 (19.4)	
ALP (U/L)	92.39 ± 49.94	100.61 ± 66.25	119.46 ± 100.21	97.63 ± 70.89	< 0.001
Albumin (mg/dL)	4.35 ± 0.42	4.31 ± 0.45	4.13 ± 0.46	4.31 ± 0.43	< 0.001
Cr (mg/dL)	0.92 ± 0.26	0.94 ± 0.48	0.92 ± 0.20	0.96 ± 0.19	0.874
Serum AFP (IU/mL)	713.13 ± 3838.31	1364.06 ± 9469.05	2285.61 ± 8267.40	718.51 ± 1428.31	0.110
PIVKA-II (mAU/mL)	755.24 ± 3340.79	1943.50 ± 8339.46	2591.83 ± 6129.87	5094.82 ± 12450.52	0.006
AST (IU/mL)	38.13 ± 27.17	41.08 ± 24.78	52.19 ± 56.47	42.08 ± 27.51	< 0.001
ALT (IU/mL)	36.30 ± 30.58	39.16 ± 27.49	46.45 ± 37.43	41.50 ± 33.77	0.009
Sum of tumor size (cm)	3.83 ± 2.26	4.26 ± 2.87	5.73 ± 3.69	5.00 ± 2.53	< 0.001
Tumor numbers	1.12 ± 0.43	1.28 ± 0.71	1.40 ± 0.94	1.34 ± 0.78	< 0.001
Pathological mUICC stage (≥ III), n (%)	75 (14.2)	86 (23.1)	54 (43.5)	14 (36.8)	< 0.001
BCLC stage (≥ C), n (%)	17 (3.2)	18 (4.8)	11 (8.9)	3 (7.9)	0.048
Beyond the Milan criteria, n (%)	110 (20.8)	94 (25.2)	58 (46.8)	18 (47.4)	< 0.001
Macrovascular invasion, n (%)	32 (6.1)	20 (5.4)	15 (12.1)	6 (15.8)	0.008

Values are presented as mean ± SD.

Abbreviations: SD, standard deviation; HCC, hepatocellular carcinoma; EHR, extrahepatic recurrence; BMI, body mass index; HBV, hepatitis B virus; HCV, hepatitis C virus; ALBI, albumin-bilirubin; ALP, alkaline phosphatase; Cr, creatinine; AFP, alpha-feto protein; PIVKA-II, prothrombin induced by vitamin K absence-II; AST, aspartate transaminase; ALT, alanine transaminase; mUICC, modified Union for International Cancer Control; BCLC, Barcelona Clinic Liver Cancer; IHR, intrahepatic recurrence.

Supplementary Table S2. Surgical findings of patients at initial HCC diagnosis (n = 1066)

Characteristic	Patients without recurrence (n = 531)	Patient with IHR (n = 373)	Patients with IHR and EHR (n = 124)	Patients without intrahepatic HCC at EHR (n = 38)	*P-value*
Margin involvement, n (%)	15 (5.3)	7 (2.8)	10 (11.2)	4 (14.8)	0.004
Microvascular invasion, n (%)	53 (10.1)	60 (16.1)	51 (41.1)	9 (23.7)	< 0.001
Serosal invasion, n (%)	8 (1.5)	6 (1.7)	8 (6.6)	3 (7.9%)	< 0.001
Bile duct invasion, n (%)	5 (1.0)	4 (1.1)	6 (5.0)	0 (0.0)	0.007
Capsule formation, n (%)	353 (68.1)	256 (70.7)	87 (71.3)	30 (78.9)	0.483
Multicentricity, n (%)	28 (5.4)	37 (10.1)	19 (15.6)	1 (2.5)	0.003
Satellite nodule, n (%)	59 (11.2)	53 (14.6)	37 (30.3)	12 (31.6)	< 0.001
Necrosis, n (%)	158 (30.3)	164 (45.3)	79 (65.3)	20 (52.6)	< 0.001
Hemorrhage, n (%)	186 (35.6)	144 (39.7)	64 (52.9)	17 (44.7)	0.005
Fatty change, n (%)	178 (34.4)	115 (31.9)	39 (32.5)	7 (18.4)	0.232
Major Edmondson–Steiner grade ≥ 3, n (%)	186 (35.2)	156 (41.8)	62 (50.0)	18 (47.4)	0.009
Worst Edmondson–Steiner grade ≥ 3, n (%)	369 (69.8)	274 (73.5)	97 (78.2)	32 (82.2)	0.077

Abbreviations: HCC, hepatocellular carcinoma; EHR, extrahepatic recurrence; IHR, intrahepatic recurrence.

Supplementary Table S3. Factors associated with EHR after surgical resection of HCC without IHR (n=1066)

Factors	Univariate analysis		Multivariate analysis	
	HR (95% CI)	*P*-value	HR (95% CI)	*P*-value
Serosal invasion	4.059 (1.246–13.230)	0.020		
Satellite nodule	2.963 (1.494–5.879)	0.002		
Macrovascular invasion	3.884 (1.611–9.362)	0.003	**3.644 (1.134–11.713)**	**0.030**
Tumor size > 5 cm	2.713 (1.431–5.142)	0.002		
Beyond Milan criteria	3.122 (1.647–5.920)	< 0.001	**3.052 (1.088–8.563)**	**0.034**
Serum PIVKA-II > 30,000 IU/mL	22.350 (4.777–4104.570)	< 0.001	**13.740 (2.594–72.778)**	**0.002**

Abbreviations: EHR, extrahepatic recurrence; HR, hazard ratio; CI, confidence interval; PIVKA-II, prothrombin induced by vitamin K absence-II; HCC, hepatocellular carcinoma.

Supplementary Table S4. Clinical findings of patients with EHR without intrahepatic HCC following surgical resection

Characteristic	Patients without intrahepatic HCC at the time of EHR (n = 38)
Neutrophil-to-lymphocyte ratio	2.33 ± 2.20
Plt ($\times 10^3$/μL)	170.56 ± 64.81
Serum AFP (IU/mL), median (range)	22.78 (0.96–12,033.04)
PIVKA (mAU/mL)	120.00 (18.0–107,763.00)
Recurrence-free survival, year (median range)	1.07 (0.2–6.87)
Location of metastasis, n (%)	
Lymph nodes	7 (17.07)
Bone	7 (17.07)
Lung	15(36.59)
Solitary/multiple	4 (9.76)/11 (26.83)
Peritoneum	5 (12.20)
Brain	2 (4.88)
Adrenal gl.	5 (12.20)
Diagnostic modality, n (%)	
Abdomen enhanced CT	22 (56.41)
Abdomen enhanced MRI	1 (2.56)
Chest X-ray	2 (5.13)
Chest enhanced CT	7 (17.95)
PET-CT	4 (10.26)
Spine MRI	1 (2.56)
Brain MRI	2 (5.13)
Survival duration after EHR (years)	2.49 (0.098-10.80)
Treatment modality after EHR	
Systemic chemotherapy	7 (18.42)
Radiotherapy	15 (39.47)
Metastatectomy	3 (7.89)
Combined therapy	8 (21.05)
Best supportive care	1 (2.63)
Follow-up loss	4 (10.53)

* Four patients exhibited multiple EHR, specifically involving the lymph nodes and peritoneum, lungs (multiple) and brain, lungs (multiple) and lymph nodes, as well as bone and adrenal glands.

Abbreviations: HER, extrahepatic recurrence; HCC, hepatocellular carcinoma; AFP, alpha-feto protein; PIVKA, prothrombin induced by vitamin K absence; CT, computed tomography; MRI, magnetic resonance imaging; PET-CT, positron emission tomography-computed tomography; Plt, platelet.

Supplementary Figures and Legends

(b)

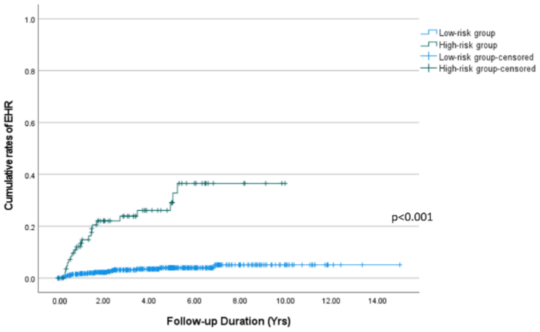

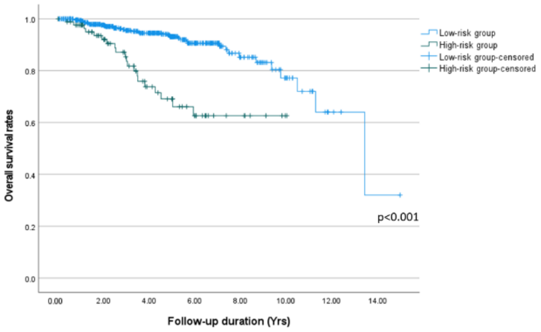

**Patients at risk**	**Total**	**1yr**	**3yr**	**5yr**	**7yr**	**10yr**	**12yr**
Low-risk group	16/482	8	13	15	16	16	16
High-risk group	22/86	10	18	20	22	22	22

**Survived patients**	**Total**	**1yr**	**3yr**	**5yr**	**7yr**	**10yr**	**12yr**
Low-risk group	482	434	298	182	82	21	4
High-risk group	86	78	48	28	8	1	0

Supplementary Figure S1. a) Cumulative EHR rates and b) overall survival rates stratified by risk group.
The cumulative EHR rates at 1, 3, 5, and 10 years were 1.7%, 3.1%, 3.9%, and 5.1%, respectively, for low-risk patients, and 12.1%, 23.9%, 29.1%, and 36.5%, respectively, for high-risk patients. The overall survival rates at 1, 3, 5, and 10 years were 99.3%, 95.5%, 93.2%, and 77.2%, respectively, for the low-risk group, and 97.6%, 85.4%, 69.1%, and 62.6%, respectively, for the high-risk group.
Abbreviations: EHR, extrahepatic recurrence.

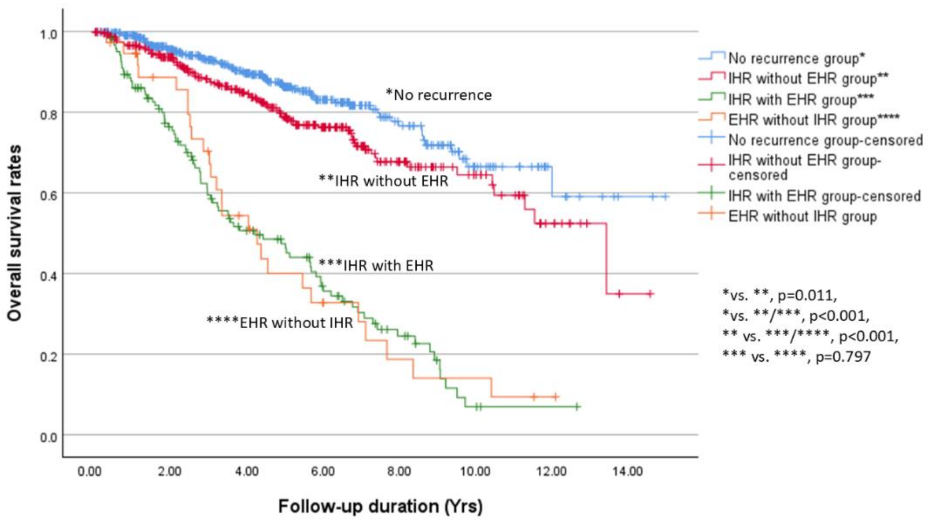

Survived patients	Total	1yr	3yr	5yr	7yr	10yr	12yr
No recurrence	531	485	330	197	94	29	9
IHR without EHR	373	341	296	168	85	30	10
IHR with EHR	124	104	84	42	22	3	0
EHR without IHR	38	35	22	11	6	3	1

Supplementary Figure S2. Overall survival rates following surgical resection of HCC stratified based on IHR and/or EHR occurrence.

Abbreviations: HCC, hepatocellular carcinoma; IHR, intrahepatic recurrence; EHR, extrahepatic recurrence.
